# Equity, multisector collaboration and innovation for rural health: lessons from the National Rural Health Association Conference

**DOI:** 10.22605/RRH8485

**Published:** 2023-06-29

**Authors:** Sagar B Dugani, Randolph D Hubach

**Affiliations:** 1 Division of Hospital Internal Medicine, Mayo Clinic, Rochester, MN, USA; and Robert D. and Patricia E. Kern Center for the Science of Health Care Delivery, Mayo Clinic, Rochester, MN, USA; 2 Department of Public Health, Purdue University, West Lafayette, IN, USA

In the US, approximately 15–20% of the population live in rural areas and face a disproportionate burden of health disparities compared with urban counterparts^1^. Resolving these challenges requires multisector infrastructure, collaboration, and innovation to evaluate and eliminate rural–urban disparities. In this Editorial, we share lessons from the National Rural Health Association’s Rural Health Equity Conference and 46th Annual Rural Health Conference in San Diego, California, on 15–19 May 2023^2,3^. As rural areas emerge from the devastating COVID-19 pandemic, the conference offered critical reminders of the unfinished business and shared responsibility in elevating US rural health and realizing the objectives of US Healthy People 2030^4^.

Equity was a major theme of the conference. The opening sessions’ speakers set the stage for problems, solutions and toolkits to address rural health inequities. In his remarks, Dr Eduardo Sanchez, Chief Medical Officer for Prevention, American Heart Association, identified several factors contributing to lagging rural health: individual health factors in rural compared to urban areas (eg tobacco use, physical inactivity), social determinants of health (eg income, education, housing, food insecurity) and healthcare system factors (eg access to outpatient and hospital care, workforce shortage). In their session, Luciana Rocha and Dr Alana Knudson from the University of Chicago Walsh Center for Rural Health Analysis discussed the Rural Health Equity Toolkit from the Rural Health Information Hub (https://www.ruralhealthinfo.org/toolkits/health-equity [https://www.ruralhealthinfo.org/toolkits/health-equity])^5^. The toolkit includes modules to frame the problem, implement and evaluate programs, and build capacity for sustainability through an equity lens. The toolkit and associated resources on general community health strategies are available for use by researchers and policymakers. Improving rural health at the individual, community and healthcare system levels will require multisector collaboration, as highlighted in presentations from the US Department of Agriculture’s Office of Rural Development, US Centers for Disease Control and Prevention, and Centers for Medicare & Medicaid Services Office of Minority Health (CMS OMH).

Improving rural health is challenging and complex, and further complicated by the availability and application of different definitions of rurality. In the US, rurality is defined at the county or subcounty level depending on the research/policy question and includes the National Center for Health Statistics urban–rural classification scheme for counties, rural–urban commuting area, and rural–urban continuum codes, among other definitions. These definitions may pose challenges, as the levels of analysis and decision-making may be different and other sociodemographic data may not be available at the preferred rurality level. This was highlighted in the presentation by the CMS OMH on applying a geographic lens and matching services and support for ‘individuals in rural, tribal, and geographically isolated communities’, with the latter including ‘frontier or remote communities, as well as the US territories and other island communities’. The speakers shared the CMS Framework for Advancing Health Care in Rural, Tribal, and Geographically Isolated Communities, which includes six priority areas including healthcare professionals, medical and communication technology, and delivery of value-based care^6^.

Several federal organizations explicitly acknowledged the need for rural-specific strategies and innovation. The Federal 2023 Omnibus Appropriations Bill included US$5 million (~A$7.5 million) to establish an Office of Rural Health at the US Centers for Disease Control and Prevention (CDC)^7^. Dr Diane Hall, Acting Director, and Dr Macarena Garcia, Senior Health Scientist and Epidemiologist, of the Office of Rural Health discussed the scope and responsibility of the Office to coordinate rural health efforts across CDC programs and improve rural–urban equity. The Office will build resources and tools and provide data that can be leveraged by researchers. The CDC speakers and others emphasized the importance of local and regional data to guide planning and decision-making. For instance, later in 2023 the CDC expects to release reports on the leading causes of early death, which could identify actionable targets at the individual and community levels and foster multisector collaboration for local interventions. The Rural Health Mapping Tool, supported by the CDC, provides county-level information on mortality, sociodemographic factors and access to health care, among other health determinants, to guide local interventions (https://ruralhealthmap.norc.org [https://ruralhealthmap.norc.org])^8^. The availability of regularly updated data will also inform progress toward the US Healthy People 2030 objectives. Availability of local data may also help with building and strengthening rural resilience, as discussed by Dr Alana Knudson, Kristine Sande and Dr Diane Hall in their session ‘Emergency preparedness and response tools’. The Rural Health Mapping Tool includes information for rural healthcare providers and policymakers to prepare for emergencies, build capacity, and promote resilience in communities and health systems. The Rural Health Mapping Tool and Rural Health Information Hub provide data on county deficits and assets (eg county social vulnerability, prosperity index score), which can provide a comprehensive rural landscape while designing and implementing strategies.

Within the past decade, the COVID-19, opioid and HIV epidemics have demonstrated that socioeconomic factors, geography, cultural context and other syndemic conditions are driving epidemics, predominantly impacting urban spaces, into rural locations where public health and healthcare resources are limited. Most national efforts to address health inequities within rural communities have taken a one-size-fits-all approach that views rural communities as homogenous entities when – in reality – rural communities are more often heterogeneous^9,10^. The sessions underscored the heterogeneity of rurality and the subpopulations making up a diverse rural America. Of note were various sessions highlighting intersectionality – the idea that, to understand inequities, categories like gender, race, where one lives (rural, urban) and socioeconomic status are best understood as overlapping and additive rather than isolated and/or distinct. Such approaches have been advocated for within the extant literature to understand inequities in rural men’s health^11^, healthy aging^12^ and in the development of a sense of community to buffer social marginalization^13,14^. Other presentations extended these discussions. For example, Dr Carrie Henning-Smith and colleagues from the University of Minnesota Rural Health Research Center discussed rural–urban differences in health for lesbian, gay, bisexual and transgender (LGBT) adults, as well as identifying challenges and best practices for supporting rural LGBT individuals. Similarly, Danielle Lucero and Dr Xavier Morales from the Praxis Project shared their experiences building a community of practice for rural community organizers among Black, Indigenous and People of Color populations.

Achieving rural health equity will require multisector collaboration to advance policy that addresses key rural health issues such as the current public health, mental health and medical workforce shortages. In the US in 2022, the Biden administration committed US$226.5 million in American Rescue Plan funding to enhance training programs for community health workers (CHWs) across the US. This cadre of health professionals, particularly in rural areas, play a critical role in connecting people to care, including COVID-19 care; mental health and substance use disorder prevention, treatment and recovery services; chronic disease care; and other important health services. The Community Health Worker Training Program is one example of bringing governmental agencies, academic institutions, community-based organizations and other stakeholders together to building a robust public health workforce. Beth Bowling and Michaela Amburgey from the University of Kentucky Center for Excellence in Rural Health provided examples of health career pipeline programs in Kentucky, including the implementation of apprenticeships for CHWs. Similarly, Floribella Redondo-Martinez and Susan Kunz from the Rural Arizona CHW Workforce Development Network described processes to building capacity of CHWs within rural organizations, while Michal Searls and colleagues from Ohio University discussed how CHWs in rural Appalachian Ohio have been leveraged to increase health literacy among community members.

## Where do we go from here?

The conference was attended by more than 1000 rural health leaders with expertise in healthcare delivery, research and policy, with a clear focus on equity, collaboration and innovation. As acknowledged by several speakers, problems and interventions may be addressed locally, but solutions may be affected by regional and federal policies. In this context, multisector collaboration and advocacy become critical parts of any rural health strategy. The responsibility to advocate rests with individuals, communities and systems at all levels to highlight challenges and, importantly, to identify opportunities and build partnerships to resolve them. As outlined by John Gale and Eric Shell in their presentations on the Future of Rural Health and The Future of Rural Healthcare, rural health faces several challenges including resetting its capabilities and infrastructure after decimation by the COVID-19 pandemic. Many rural communities are threatened by rural hospital closure^15^ and workforce shortage^16^, and require targeted strategies for recovery, sustenance and resilience. The conference reinforced that action to improve rural health will require multisector collaboration from philanthropy, non-governmental organizations, governmental organizations, researchers, community organizations and others to ensure that people’s basic needs are met (eg housing, food), while also preparing neighborhoods (eg safe walking areas) and health systems (eg staffing) to transform rural health. In order to do this, we will require better, local data to evaluate and monitor progress toward local, regional, and federal health targets. While focusing on basic infrastructure, processes and systems that support overall health and care, it will be imperative to also focus on specific health conditions (eg mental health, the opioid crisis), which have been particularly devastating for rural communities.

Lessons from these conferences are pertinent to rural populations beyond the US. An estimated 50% of the world’s population live in rural communities and, similarly to the US, experience various health disparities^17–19^. In 2021, a consortium of six organizations developed a global manual to define cities, towns and rural areas for international comparison^20^. This will facilitate international studies, permit comparison and allow for the identification of common themes affecting rural health. Notwithstanding the challenges for international studies, strategies to address workforce shortage, as well as inadequate funding, multisector collaboration in the US may be adapted to other countries. In turn, lessons from other countries will shape strategies and tactics in the US. This will allow for a dynamic exchange of information, knowledge and experience, which will elevate rural health globally.

Rural health and care are at an inflection point. Concerted multisector collaboration can direct attention toward problems and transformative solutions to advance health for people in rural areas. Rural health is public health, and the time to act is now. We cannot achieve health equity without advancing rural health.
